# Generation of multi-gene knockout rabbits using the Cas9/gRNA system

**DOI:** 10.1186/2045-9769-3-12

**Published:** 2014-09-27

**Authors:** Quanmei Yan, Quanjun Zhang, Huaqiang Yang, Qingjian Zou, Chengcheng Tang, Nana Fan, Liangxue Lai

**Affiliations:** 1 Key Laboratory of Regenerative Biology, South China Institute for Stem Cell Biology and Regenerative Medicine, Guangzhou Institutes of Biomedicine and Health, Chinese Academy of Sciences, Guangzhou, 510530 China; 2 College of Animal Science, Jilin University, Changchun, 130062 China

**Keywords:** Cas9/gRNA system, Rabbits, Multiple-gene knockout

## Abstract

The prokaryotic clustered regularly interspaced short palindromic repeat (CRISPR)-associated system (Cas) is a simple, robust and efficient technique for gene targeting in model organisms such as zebrafish, mice and rats. In this report, we applied CRISPR technology to rabbits by microinjection of Cas9 mRNA and guided RNA (gRNA) into the cytoplasm of pronuclear-stage embryos. We achieved biallelic gene knockout (KO) rabbits by injection of 1 gene (IL2rg) or 2 gene (IL2rg and RAG1) Cas9 mRNA and gRNA with an efficiency of 100%. We also tested the efficiency of multiple gene KOs in early rabbit embryos and found that the efficiency of simultaneous gene mutation on target sites is as high as 100% for 3 genes (IL2rg, RAG1 and RAG2) and 33.3% for 5 genes (IL2rg, RAG1, RAG2, TIKI1 and ALB). Our results demonstrate that the Cas9/gRNA system is a highly efficient and fast tool not only for single-gene editing but also for multi-gene editing in rabbits.

**Electronic supplementary material:**

The online version of this article (doi:10.1186/2045-9769-3-12) contains supplementary material, which is available to authorized users.

## Introduction

Mice have long been used as animal models for gene function studies and human diseases. However, due to differences in the physiological traits and gene expression between mice and humans, mouse models cannot replicate the symptoms or pathology of human diseases in some cases [1]. For example, a recent study indicated that the genomic responses of inflammation in mouse models correlate poorly with human conditions [2]. Thus, animal models more similar to humans that can mimic complex human conditions are urgently needed for translational medical research.

Rabbits have more similarities with human beings in terms of physiology, anatomy and genetics than mice and rats [3]. Compared with large animals such as pigs and monkeys, rabbits have a shorter gestation term and require lower maintenance cost. All these advantages make rabbits a more appropriate animal model for many diseases, such as cardiovascular and metabolic diseases. However, prior to the advent of newly emerging gene-editing technologies, such as custom-made zinc-finger nucleases (ZFNs) [4] and transcription activator-like effectors nucleases (TALENs) [5], gene editing in rabbits was difficult because the ability of rabbit embryonic stem cells (ESCs) or induced pluripotent stem cells (iPSCs) to contribute to germ line transmission has not been successfully established and the efficiency of somatic cell nuclear transfer (SCNT) in rabbits is much lower than in other animals such as cattle, pigs, sheep and mice [6]. Thus far, only a few successful cases of gene-targeting rabbits have been reported.

Gene KO rabbits were successfully produced using ZFN and TALEN technologies in 2011 and 2012, respectively [3, 7, 8]. However, the possibility of producing multi-gene KO rabbits using ZFNs and TALENs has not been reported. In many cases, multiple genes must be simultaneously knocked out in rabbits, given that many diseases are caused by alteration of more than 1 gene, in order to study gene interactions.

The latest gene-targeting technology, CRISPR/Cas system [9, 10], has been successfully applied to edit genes in Drosophila [11, 12], C. elegans [13], plants [14], zebrafish [15–17], mouse [18], rat [19, 20], livestock cells [21], human cells [22, 23], monkey [24] and rabbit [25]. The Cas9/gRNA system is developed by fusing 2 small RNA (CRISPR RNA and transactivating crRNA) to form a gRNA, which guides the Cas9 protein to cleave specific DNA [23, 26]. The mechanism of Cas9/gRNA system for editing specific genes is similar to ZFNs and TALENs [3]: double-strand breakss are created in the targeted sites, after which several base deletions or insertions (indels) are brought in by non-homologous end-joining; if a donor DNA homologous to the flanks of the double-strand breaks is offered, precise DNA fragment replacement will occur through homology-directed repair. Although the techniques share similar mechanisms, the CRISPR/Cas system has several advantages over ZFNs and TALENs. The CRISPR/Cas system, for example, offers higher efficiency and easier design steps. More importantly, it can potentially target multiple genes in a single step because multiple gRNAs can share the same endonuclease Cas9 for multiple gene-KO [18, 27]. Two gene-KO mice and 3 gene-KO rats have been obtained by one step injection of Cas9 mRNA and gRNA to zygotes [18, 20].

In this report we applied CRISPR technology to rabbit by microinjection of Cas9 mRNA and gRNA into the cytoplasm of pronuclear-stage embryos to test the gene editing efficiency of the technique and explore the feasibility of generating multiple gene-KO in rabbits in a single step.

## Results

### The Cas9/gRNA system

**Figure 1:**
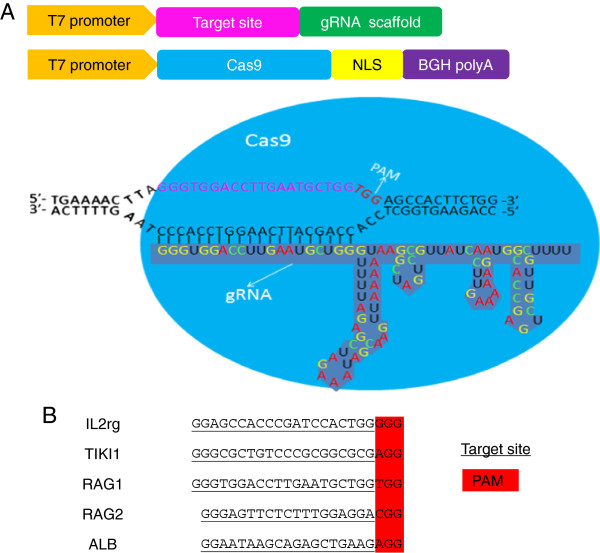
**Genome editing via the Cas9/gRNA system in rabbit. (A)** Constructs and schematic illustration of the Cas9/gRNA system used in this study. The T7 promoter drives transcription of the gRNA, consisting of a target sequence and a scaffold sequence. NLS: nuclear localization signal. bGH polyA: bovine growth hormone poly-A. **(B)** Target sequence of the IL2rg, RAG1, RAG2, TIKI1 and ALB genes using the Cas9/gRNA system. gRNA-targeting sequences are underlined and PAM sequences are highlighted in red.

We constructed a vector with the T7 promoter that can be *in vitro* transcribed into Cas9 mRNA and gRNA (Figure 1A). The sequences of all target sites (22 nucleotides or 23 nucleotides) (Figure 1B) begin with GG at the 5’ end because transcription is initiated from GG bases and ended with the protospacer adjacent motif (PAM) NGG at the 3’ end, which is indispensable for Cas9 binding and cleavage [27].

### Generation of 1 gene-KO rabbits by Cas9/gRNA system

**Figure 2:**
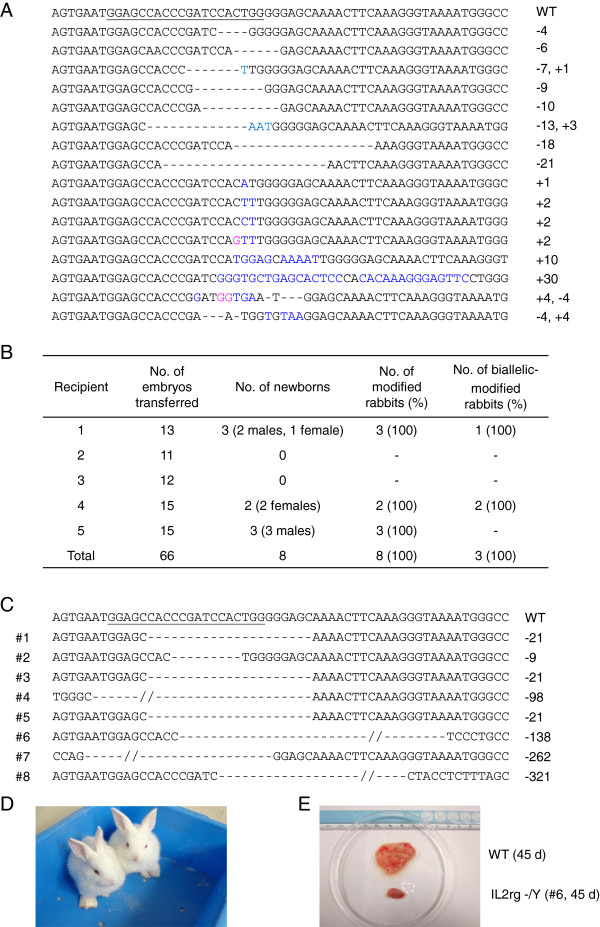
**One gene (IL2rg)-KO rabbit embryos and newborn rabbits. (A)** Sequenced mutations in the IL2rg gene in injected embryos. Deletions are indicated by dashes, insertions are indicated in blue and substitutions are indicated in pink. **(B)** Generation of IL2rg KO rabbits via the Cas9/gRNA system. Zygotes (n = 66) microinjected with 200 ng/μL of Cas9 mRNA and 20 ng/μL of gRNA for IL2rg were transferred to 5 recipient mothers, 3 of which gave birth to 8 live kits. **(C)** Detailed mutations of the IL2rg gene in the 8 KO founders. The number of founder KO rabbits is shown in the left column. **(D)** Picture of 26 days old IL2rg KO rabbits. **(E)** The thymus of IL2rg KO rabbits was obviously smaller than that from age-matched WT ones. In A and C, the WT sequence is shown at the top with the target sites in underline; the sizes of the deletions (-) or insertions (+) are shown in the right column. At least 8 TA-clones for each embryos or KO rabbit were used for sequencing to obtain detailed information of the mutation.

We selected the IL2rg gene (X-linked) as the first gene of interest to test the efficiency of 1 gene-KO rabbits. A mixture of Cas9 mRNA and gRNA for IL2rg was microinjected into the cytoplasm of pronuclear-stage embryos with working concentrations of 200 ng/μL of Cas9 mRNA and 20 ng/μL of gRNA. Sixteen of 21 injected embryos developed into the blastocyst stage, and PCR products derived from 12 blastocysts were sequenced to confirm mutation efficiency. As shown in Table 1, IL2rg mutation was found in all 12 embryos. More strikingly, we detected no wild type (WT) sequence among the 12 embryos. The indels of IL2rg gene ranged from 30 base pair (bp) insertions to 21 bp deletions (Figure 2A). We then used this system to produce IL2rg KO rabbits. A total of 66 embryos injected with the same concentration of Cas9 mRNA and gRNA for IL2rg were transferred to 5 pseudo-pregnant recipient rabbits. After about 1 month, 3 of 5 recipient mothers were pregnant to term and gave birth to 8 live kits (5 males, 3 females) (Figure 2B). PCR-sequencing of the targeted site in rabbits showed that all 5 male newborns were IL2rg KO in X chromosome and all 3 female newborns were mutated in both X chromosomes. The indels in the founders ranged from 9 bp to 321 bp deletions (Figure 2C). All IL2rg KO rabbits were alive for no more than 45 days because of diarrhea, pulmonary infection or other causes, except the #2 (female, lived for 229 days) rabbit, kept in conventional housing conditions (Figure 2D). We performed the autopsies soon after their death and found that IL2rg KO rabbits had undersized thymuses compared with age-matched WT ones (Figure 2E).

**Table 1:** ***In vitro*** **development of embryos injected with Cas9 mRNA and gRNAs knocking out a single gene**

Gene	gRNA/Cas9 mRNA (ng/μL)	No. of injected zygotes	No. of blastocysts (%)	No. of sequenced blastocysts	No. of modified (%)	No. of biallelic- modified (%)
IL2rg	20/200	21	16 (76)	12	12 (100)	12 (100) *
TIKI1	20/200	10	8 (80)	8	8 (100)	4 (50)

*Indicates embryos without detecting WT sequence.

**Figure 3:**
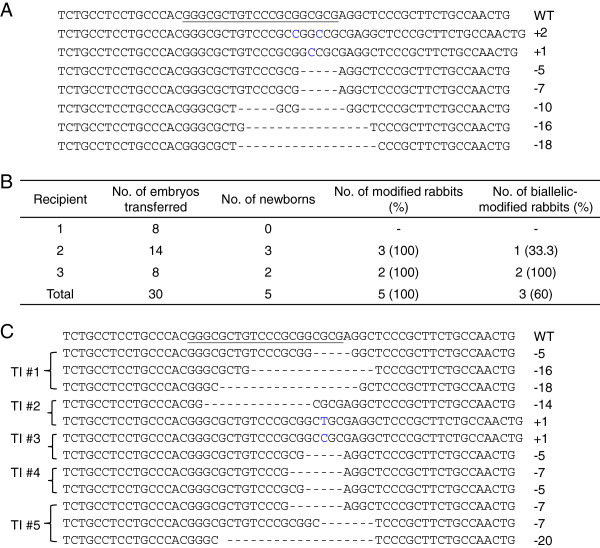
**One gene (TIKI1)-KO rabbit embryos and newborn rabbits. (A)** Sequenced mutations in the TIKI1 gene in injected embryos. Deletions are indicated by dashes and insertions are indicated in blue. **(B)** Generation of TIKI1 KO rabbits by the Cas9/gRNA system. The zygotes (n = 30) microinjection with the 200 ng/μL of Cas9 mRNA and 20 ng/μL of gRNA for TIKI1 were transferred to 3 recipient mothers, 2 of which gave birth to 5 live kits. **(C)** Detailed mutations of the TIKI1 gene in the 5 KO founders. The number of founder KO rabbits is shown in the left column. In A and C, the WT sequence is shown at the top with the target sites in underline, and the sizes of the deletions (-) or insertions (+) are shown in the right column.

To further confirm high gene KO efficiency in rabbits by the Cas9/gRNA system, we repeated the experiment with the same approach for another gene, TIKI1 [28]. For *in vitro* testing, we microinjected 10 pronuclear-stage embryos with Cas9 mRNA and gRNA for TIKI1 at the same concentration as IL2rg. A total of 8 blastocysts were obtained and sequenced. Consistent with the results of IL2rg, the modification efficiency was also 100% (8/8) amongst the embryos, and 4 of the 8 embryos were modified in both alleles (50%) (Table 1). The indels of the TIKI1 in the injected embryos ranged from 2 bp insertions to 18 bp deletions (Figure 3A). Thirty embryos injected with 200 ng/μL of Cas9 mRNA and 20 ng/μL of gRNA for TIKI1 were transferred to 3 pseudo-pregnant recipient rabbits. After about 1 month, 2 of 3 recipient mothers were pregnant to term and gave birth to 5 live kits (Figure 3B). PCR-sequencing of the targeted site in rabbits showed that all 5 newborns were modified at the TIKI1 gene site and that 3 out of 5 newborns were biallelically modified. The indels in the founders ranged from 1 bp insertions to 20 bp deletions (Figure 3C). The #1, #2, and #3 TIKI1 KO rabbits were weak at birth and died within 3 days. The #4 and #5 TIKI1 KO rabbits have been alive for over 5 months with apparent normal phenotype and would require further study.

### One step generation of 2 gene-KO rabbits by Cas9/gRNA system

**Figure 4:**
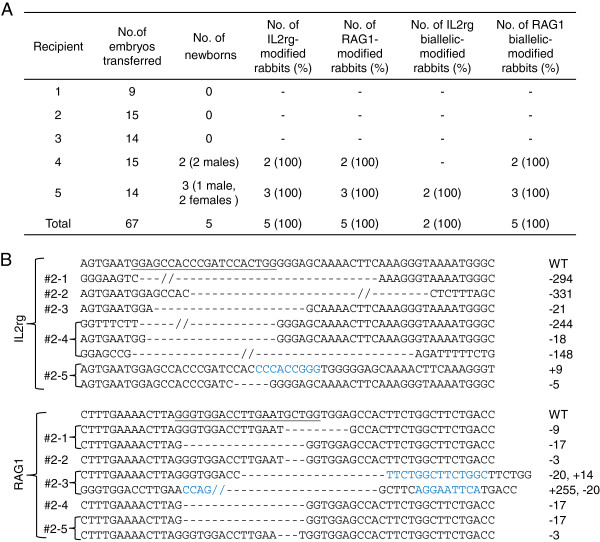
**Two gene-KO rabbits obtained by the Cas9/gRNA system in a single step.** **(A)** Generation of 2 gene-KO (IL2rg/RAG1) rabbits via the Cas9/gRNA system. Zygotes (n = 67) were microinjected with 200 ng/μL of Cas9 mRNA, 20 ng/μL of gRNA for IL2rg and 20 ng/μL of gRNA for RAG1 and transferred into 5 recipient mothers, 2 of which gave birth to 5 live kits. **(B)** Detailed mutations of the IL2rg and RAG1 genes in the 5 KO founders. The number of founder KO rabbits is shown in the left column. For each gene, the WT sequence is shown at the top with the target sites in underline, deletions are indicated by dashes and insertions are indicated in blue, and the sizes of the deletions (-) or insertions (+) are shown in the right column.

We tested the efficiency of 2 gene modification in 1 step using the Cas9/gRNA system in rabbits. IL2rg and RAG1 were chosen as the genes of interest. Rabbits with a deficiency of IL2rg and RAG1 genes are expected to be an important immunodeficient animal model without mature T cells, B cells and NK cells [29]. A total of 67 zygotes co-injected with a mixture of Cas9 mRNA (200 ng/μL), gRNA for IL2rg (20 ng/μL) and gRNA for RAG1 (20 ng/μL) were transferred into 5 surrogate rabbits. After about 1 month of pregnancy, 2 out of 5 recipient mothers developed to term and gave birth to 5 live kits (3 males, 2 females) (Figure 4A). DNA sequencing of the PCR products spanning the targeted site showed that both IL2rg and RAG1 genes were simultaneously modified in all 5 newborns. The RAG1 gene was biallelically modified in all 5 rabbits and IL2rg gene biallelically modified in both female rabbits. Two of the 5 kits carried the same mutation pattern in 2 alleles (homozygous mutation) at the RAG1 locus. Mutations of the IL2rg gene in the founders ranged from 8 bp insertions to 331 bp deletions (Figure 4B, upper), whilst those of the RAG1 gene ranged from 246 bp insertions to 20 bp deletions (Figure 4B, lower). The #1, and #2 IL2rg/RAG1 KO rabbits were weak at birth and died within 2 days. The other 3 IL2rg/RAG1 KO rabbits died within 2 months (because of infection) when kept in cleaner conditions in isolation after weaning. The thymus phenotype of these KO rabbits was similar to IL2rg KO rabbits (data not shown).

### Simultaneous multi-gene KO *in vitro*rabbit embryos by Cas9/gRNA system

We co-injected a mixture of 3 gRNAs targeting IL2rg, RAG1 and RAG2 plus Cas9 mRNA into the cytoplasm of pronuclear-stage embryos to test the feasibility of creating multi-gene KO rabbits. The working concentrations were 20 ng/μL for each gRNA and 200 ng/μL for Cas9 mRNA. Out of 24 injected embryos, 20 embryos developed into the blastocyst stage (83.3%). All 20 blastocysts were used for PCR and sequence tests. We successfully obtained sequence data for all 3 genes in 16 of the blastocysts. All 16 embryos (100%) showed triple-gene mutations and 15 (93.8%) did not show WT sequences for all 3 genes (Table 2). As shown in Additional file 1: Figure S1A, the indels ranged from 3 bp insertions to 22 bp deletions for the IL2rg gene, 18 bp insertions to 9 bp deletions for the RAG1 gene and 1 bp insertions to 15 bp deletions for the RAG2 gene.

**Table 2:** ***In vitro*** **development of embryos injected with Cas9 mRNA and gRNAs knocking out 3 genes simultaneously**

Gene	gRNA/cas9 mRNA (ng/μL)	No. of injected zygotes	No. of blastocysts (%)	No. of sequenced blastocysts	No. of modified (%)	No. of biallelic-modified (%)	No. of 2 gene- modified (%)	No. of 3 gene- modified (%)	No. of 3 gene biallelic-modified (%)
IL2rg				18	18 (100)	18 (100)*			
RAG1	20/20/20/200	24	20 (83.3)	19	19 (100)	17 (89.5)	18/18 (100)	16/16 (100)	15/16 (93.75)*
RAG2				16	16 (100)	16 (100)			

*indicates embryos without detecting WT sequence.

The high efficiency of successful targeting of 3 genes in *in vitro* cultured embryos encouraged us to determine whether or not the Cas9/gRNA system has the capacity to knock out more genes simultaneously in 1 step. We microinjected a mixture of 5 gRNAs targeting IL2rg, RAG1, RAG2, TIKI1 and ALB combined with Cas9 mRNA into pronuclear embryos. The working concentrations were 20 ng/μL for each gRNA and 200 ng/μL for Cas9 mRNA. Out of 39 injected embryos, 20 developed into blastocyst stage (51.3%), lower than that in the 3 gene-KO group. We successfully obtained sequence data for all 5 genes in 12 embryos. As shown in Table 3, 1 to 5 mutations could be found in an individual embryo and 4 sequenced embryos were discovered with 5 genes edited simultaneously (33.3%). More interestingly, 1 embryo did not show WT sequences for all 5 genes. The detailed mutation patterns of the 5 genes are presented in Additional file 1: Figure S1B.

**Table 3:** ***In vitro*** **development of embryos injected with Cas9 mRNA and gRNAs knocking out simultaneously 5 genes**

Gene	gRNA/cas9 mRNA (ng/μL)	No. of injected zygotes	No. of blastocysts (%)	No. of sequenced blastocysts	No. of modified for a single gene (%)	No. of biallelic- modified (%)	No. of 2 or more genes- modified (%)	No. of 3 or more genes- modified (%)	No. of 4 or more genes- modified (%)	No. of 5 genes- modified (%)	No. of 5 genes biallelic-modified (%)
IL2rg				12	11 (91.7)	11 (91.7) *					
RAG1				15	14 (93.3)	12 (80.0)					
RAG2	20/20/20/20/20/200	39	20 (51.3)	14	12 (85.7)	10 (71.4)	11/12 (91.7)	7/12 (58.3)	6/12 (50.0)	4/12 (33.3)	1/12 (8.33)*
TIKI1				14	9 (64.3)	6 (42.9)					
ALB				19	11 (57.9)	5 (26.3)					

*indicates embryos without detecting WT sequence.

### Off-target analysis of newborn rabbits

**Figure 5:**
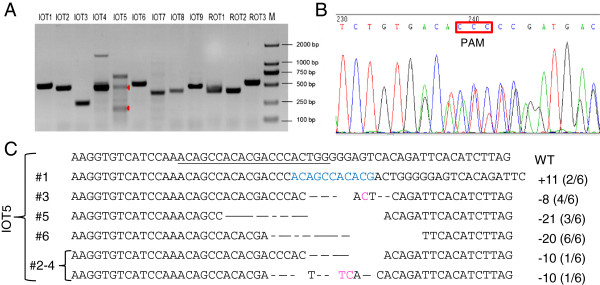
**Off-target analysis of Cas9/gRNA system-induced mutations in KO founders. (A)** T7EI assays of the PCR products of candidate off-target sites using the pooled DNA of all KO founders for each gene as the template (primer sequences listed in Additional file 3: Table S2). The IOT5 could be cleaved (red arrowheads). **(B)** Sequencing diagram of IOT5 in #1 IL2rg KO new-born rabbits showing a double curve after the mutation around the PAM region. **(C)** Detailed mutations of IOT5 in the newborn rabbits. The number of founder KO rabbits is shown in the left column. The WT sequence is shown at the top with the target sites in underline. Deletions are indicated by dashes, insertions are indicated in blue and substitutions are indicated in pink, and the sizes of the deletions (-) or insertions (+) are shown in the right column. The fractions indicate the read number of the mutant allele (numerator) out of total read number (denominator).

Several studies have suggested that the 8–12 bases (seed sequence) of the gRNA sequence close to PAM and PAM itself are critical factors determining site-specific cleavage. In other words, slight mismatches beyond the seed sequence may lead to off-target cleavage [27]. To detect the occurrence of off-target mutations in all 18 newborn rabbits (8 IL2rg KO rabbits, 5 TIKI1 KO rabbits and 5 IL2rg/RAG1 KO rabbits), we selected candidate sites with over 14 bases identical to the targeted sites of IL2rg, TIKI1 and RAG1. Nine sites (IOT1 to IOT9) were determined to be potential off-targeting sites for the IL2rg gene, 17 sites (TOT1 to TOT17) were determined to be potential off-targeting sites for the TIKI1 gene, whilst 3 sites (ROT1 to ROT3) were determined to be potential off-targeting sites for the RAG1 gene in the rabbit genome (Additional file 2: Figure S2). T7 endonuclease I (T7EI) assay showed that only the IOT5 site presented detectable mutagenic activity amongst these 29 potential off-target sites in all founder rabbits (Figure 5A). To confirm the results of T7EI assay, we further examined the DNA sequences of PCR products. Mutations at the IOT5 site occurred around the PAM region (Figure 5B). DNA sequencing showed that 5 (#1, #3, #5, #6, #2-4) of 13 founders had mutations at the IOT5 site. Details of these mutations are shown in Figure 5C.

## Discussion

In this report, we describe the feasibility of applying the Cas9/gRNA system to create multi-gene KO rabbits. In the current work, we first applied the Cas9/gRNA system to generate single gene-KO rabbits (IL2R or TIKI1). We found that the efficiency of single gene modification is as high as 100% in both *in vitro* embryos and newborn rabbits. In our previous study using TALEN technology to generate KO rabbits, we were unable to achieve such a high efficiency [8]. The mutation patterns derived from Cas9/gRNA technology included chimera with at least 2 different genetic modifications, biallelic mutation and homozygote, similar to that using TALENs [8]. However, on account of its easy design, low time consumption and reduced financial cost, the Cas9/gRNA system is superior to TALENs. To construct gRNA vectors for specific genes, the targeting sequences can be determined by simply selecting the sequences with GGN18NGG or GGN17NGG (target sites are 19 nucleotides or 20 nucleotides or even shorter like 17 nucleotides and 18 nucleotides [30]). The oligonucleotides can be artificially synthesized and annealed into a double-strand DNA with cohesive terminus, which then can be cloned into the gRNA vector. The whole process can be completed within 2 days.

Encouraged by the high efficiency of the Cas9/gRNA system for editing 1 gene, we injected 2 gRNAs targeting IL2rg and RAG1 and Cas9 mRNA into one-cell stage embryos to test the efficiency of the system for 2 gene-KO in a single step. Results indicated that all 5 newborns were simultaneously modified at the IL2rg and RAG1 genes. Moreover, the RAG1 gene was biallelically modified in all 5 kits and IL2rg was modified in both alleles of all 2 female kits, consistent with the findings in mice [18]. More interestingly, we also found that 1 of these rabbits carried the same mutation at the same locus in the 2 alleles, a result that is similar to homozygotes created by traditional homologous recombination. This finding has not been described in similar work with mice [18].

Because site-specific cleavage depends on gRNA and all gRNAs share the same Cas9 enzyme, we speculated that more than 2-gene KO rabbits may be generated by co-injecting several gRNAs together with Cas9 mRNA into pronuclear-stage rabbit embryos. We explored this feasibility using *in vitro* developmental rabbit embryos. We co-injected a mixture of 3 gRNAs combined with Cas9 mRNA into the cytoplasm of pronuclear-stage embryos using a similar methodology employed in rats [20]. The efficiency of 3 gene-KO was also highly favourable: all 15 embryos tested had 3 gene mutations and all 3 genes were biallelically mutated simultaneously. We then extended the number of injected gRNAs to 5. To date, a similar trial has yet to be reported in rabbits or other mammals. A variety of mutations (1 to 5) were observed. Four embryos (33.3%) had 5 genes knocked out simultaneously. This efficiency is favorable enough for practical application in the generation of multi-gene KO rabbits. The *in vitro* development of embryos was not substantially affected since there was 83% survival to blastocyst in the 3 gRNA-injected embryos, while there was a modest effect for the 5 gRNA-injected embryos (51.3% survival to blastocyst). In our previous studies using TALEN technology we achieved 36% survival to blastocyst for RAG2 [8] and in the study using the Cas9/gRNA system by Yang *et al.* there was 49.2% survival to blastocyst for APOE [25]. Therefore, achieving KO rabbits with mutations of 5 genes in 1 step might be also feasible when transferring targeted embryos into surrogates.

Off-targeting is a major concern in gene editing technology, and analysis of off-target events in the founder animals is a necessity. Several previous studies have shown that the seed sequence is a key factor in determining the targeting specificity of the Cas9/gRNA system. Off-targeting is more commonly observed when the matched sequences are located closer to the PAM [27]. In our study, off-target mutations were found in 5 out of 13 founders and only occurred at the IOT5 site. However, this site is supposed to be a less likely candidate for off-target effects than the IOT1, IOT3 and IOT4 sites. The reason behind this event is unknown. However, mutations at IOT5 did not affect the birth and phenotype of IL2rg KO rabbits. There are many reported strategies to reduce off-targets of the Cas9/gRNA system, such as the ‘paired nicking’ system [31] or truncated gRNA [30]. Reducing the concentration of injected gRNA and Cas9 mRNA may also decrease the efficiency of off-targets, but at the expense of decreasing cleavage on on-target site [32].

Besides zygote microinjection to generate KO rabbits, SCNT and ESCs or iPSCs might also be applied to generate KO rabbits. However, the ability of rabbit ES cells or iPS cells to contribute to germ line transmission has not been successfully established and the efficiency of SCNT in rabbits is much lower. So it is a long way to go before using ESCs or iPSCsand SCNT to generate KO rabbits, and zygote microinjection is a relatively easy technology to generate KO rabbits.

The IL2rg-KO rabbits generated in this study will be used for experiments like cell transplantation therapy and xenotransplantation, which maybe useful to bridge the gap between small animals and large animals.

While this manuscript was in preparation, Yang *et al.* reported the Cas9/gRNA system was feasible to edit genes in rabbits. However, the average efficiency of 1 gene-KO in rabbits in our study is higher than that in this report (100% compared to 52.6% in rabbits embryos and 100% to 55.9% for newborn rabbits), and we knocked out multiple genes at a time while they just knocked out 1 gene at a time. Moreover, Yang *et al.* did not analyze the off-targets.

In conclusion, the Cas9/gRNA system is a highly efficient and fast tool for generating gene KO in rabbits and can be utilized to construct multiple gene modifications in 1 step. Application of the Cas9/gRNA system will greatly promote genetic engineering of rabbits, which will aid in determining gene functions and establishing valuable modified-animal models for human disease research.

## Methods

### Animals

The rabbits used in this study were New Zealand rabbits strain and were purchased from the Laboratory Animal Centre of Southern Medical University. Animal experiments were approved by the Animal Research Ethics Committee of the Guangzhou Institutes of Biomedicine and Health, Chinese Academy of Sciences.

### gRNA design

Sequences of GGN18NGG or GGN17NGG in the sense or antisense strand of the DNA were selected as the target sites. Two DNA oligos (s1: 5’-ATAGGN18 [or N17] GT-3’; s2: 5’-TAAAC plus the reverse complement of GGN18 [or GGN17]-3’ ) were synthesized for each targeted site. The 2 DNA oligos (10 μM) were annealed in New England Biolabs (NEB) buffer 3 under previously described conditions [16]. A mixture of 4 μL of annealed solution, 5 μL of solution I (Takara) and 1 μL of purified gRNA cloning vector (Figure 1A) digested with Bbs I (Thermo) was maintained at 16°C for 30 minutes and then transformed into competent bacteria for plasmid preparation and subsequent sequencing analysis to select the correct gRNA containing the target site sequence.

### RNA synthesis

The Cas9 expression vector (from Addgene) was linearized with Pme I (Thermo) and purified with an agarose gel DNA extraction kit (Takara). The Cas9 mRNA was transcribed with mMESSAGE mMACHINE^®^ T7 Kit (Ambion), poly (A) tailed with *E. coli* Poly (A) Polymerase (NEB) and then purified with lithium chloride precipitation following the manufacturer’s protocol.

The templates of gRNA for *in vitro* transcription of RNA were purified PCR products obtained from gRNA vectors using the primer pair (T7-F: 5’-GAAATTAATACGACTCACTATA-3’ and T7-R: 5’-AAAAAAAGCACCGACTCGGTGCCAC-3’) with a high-fidelity enzyme (Takara). The gRNA was transcribed using a T7 High Yield RNA Synthesis Kit (NEB) and purified by lithium chloride precipitation following the manufacturer’s protocol.

The quality of the synthesised RNA was analysed by 1% agarose gel electrophoresis at 200 V for 8 minutes, and its concentration was determined by spectrophotometry. All of the reagents used in the experiments above were RNase-free.

### Collection of rabbit zygotes

Zygotes were collected from superovulated donor rabbits as previously described [8]. Briefly, sexually mature rabbits were injected with 100 IU pregnant mare serum gonadotrophin intramuscularly. About 72 hours later, the rabbits were mated and then injected with 100 IU human chorionic gonadotrophin (hCG) intravenously. The day after mating (about 10 hours to 20 hours later), donor rabbits were sacrificed and zygotes were flushed from the oviducts with pre-warmed embryo manipulation medium (M199). Pronuclear-stage zygotes were transferred to embryo culture medium for microinjection.

### Microinjection of the Cas9 mRNA and gRNA mixture into zygotes

The final concentration of each gRNA in the mixture solution was 20 ng/μL, and the Cas9 mRNA concentration was 200 ng/μL. Approximately 5–10 pL of the mixture liquid was injected into the cytoplasm of pronuclear-stage embryos using an injection pipette on a heated microscope stage set at 37.5°C. The injected embryos were transferred to embryo culture medium for either about 5 days to blastocyst stage for *in vitro* embryo development testing or 2–3 hours for embryo transfer.

### Embryo transfer

Detailed manipulations of embryo transfer were as described by Tian *et al.* [33]. Briefly, the recipient mothers were injected with 100 IU hCG at the same day the donor rabbits were injected with 100 IU hCG. About 8 embryos were injected into the unilateral pavilion of oviduct (16 embryos for bilateral pavilion of oviduct) for each recipient mother.

### Mutation detection of targeted genes in embryos and newborns

Each blastocyst or morula embryo was individually collected in a PCR tube. The DNA of single embryos was extracted with 6 μL of embryo lysis buffer (0.45% NP40 plus 60 ng/μL protein K in double distilled water at 50°C for 20 minutes and 90°C for 5 minutes in a BIO-RAD thermal cycler. The DNA of new-born rabbits was extracted from a small piece of ear tissue using a DNA extraction kit (Takara) following the manufacturer’s protocol. PCR products spanning the target sites were amplified with Premix Tag Polymerase (Takara) under the following conditions: 95°C for 3 minutes, 35 cycles of 94°C for 30 seconds, 60°C for 30 minutes and 72°C for 30 seconds or 1 minutes, 72°C for 5 minutes and 4°C for an indeterminate period. Sequencing followed.

We performed nested PCR to simultaneously test 5 genes in a single embryo with 25 cycles during first PCR run and 35 cycles during the second PCR run. All primers used are listed in Additional file 3: Table S1. The PCR products showing a different curve compared with those of WT animals were cloned into the pMD-18 T vector (Takara). At least 8 TA-clones selected from each transformation were used for sequencing to obtain detailed information of the mutation.

### Off-target analysis of newborn rabbits

Sites with over 14 bp identical to the sequence of gRNA (20 bp) and NGG (PAM, 3 bp) in the rabbit genome were selected as candidate off-target sites. Nine such sites was selected and screened for the IL2rg gene in 8 IL2rg KO rabbits and 5 IL2rg/RAG1 KO rabbits, 17 such sites were screen for TIKI1 in 5 TIKI1 KO rabbits, whilst 3 such sites were screened for RAG1 in 5 IL2rg/RAG1 KO rabbits. The primers used to amplify the candidate off-target sites are listed in Additional file 3: Table S2.

### T7E1 assay

PCR products spanning the potential off-target sites were amplified using the DNA pool of all new-born rabbits as the template. A mixture of 9 μL of PCR products and 1 μL of NEB buffer 2 was melted and re-annealed in a thermal cycler under the following conditions: 95°C for 10 minutes, 95°C to 85°C at 2°C/second, 85°C for 1 minute, 85°C to 25°C at 0.1°C/second, 25°C for 1 minute and 4°C for an indefinite period. Exactly 6 μL of the re-annealed mixture was treated with 0.5 uL of T7 endonuclease I (5 units) by addition of 0.4 μL of NEB buffer 2 and 3.1 μL of dd H_2_O at 37°C for 20 minutes. Analysis by 2% agarose-gel electrophoresis followed. The gel was stained with 1 μg/mL ethidium bromide in Tris-acetate-EDTA buffer for 5 minutes and then imaged with a gel-imaging system.

## Electronic supplementary material

Additional file 1: Figure S1: Detailed mutations of muti-gene KO in rabbit embryos (A) Sequenced mutations of the IL2rg, RAG1 and RAG2 genes in *in vitro* developmental embryos by microinjected of Cas9 mRNA together with gRNAs for IL2rg, RAG1 and RAG2. (B) Sequenced mutations of the IL2rg, RAG1, RAG2, TIKI1 and ALB genes occurred in *in vitro* developmental embryos by microinjection of Cas9 mRNA together with gRNAs for IL2rg, RAG1, RAG2, TIKI1 and ALB. For each gene, the WT sequence is shown at the top with the target sites in underline. Deletions are indicated by dashes, insertions are indicated in blue and substitutions are indicated in pink, and the sizes of the deletions (-) or insertions (+) are shown on the right column. (PPTX 85 KB)

Additional file 2: Figure S2: Candidate off-target sites are presented in terms of chromosome location, sequence, overall percent match with the targets (mismatches are indicated in red) and expected amplicon and T7EI fragment sizes. (PPTX 113 KB)

Additional file 3: Table S1: Primer pairs used to amplify the fragments encompassing the targeted sites of IL2rg, RAG1, RAG2, TIKI1 and ALB. Table S2. Primer pairs used to amplify the fragments encompassing the candidate off-targeted sites of IL2rg, RAG1and TIKI1. (DOC 82 KB)
