# Supplementary material for: Generation of multi-gene knockout rabbits using the Cas9/gRNA system
Source: Cell Regen. 2014 Sep 27;3:12. doi: 10.1186/2045-9769-3-12 (PMC4230364; doi:10.1186/2045-9769-3-12)
Supplement: Supplementary file 1 — Additional file 1: Figure S1: Detailed mutations of muti-gene KO in rabbit embryos (A) Sequenced mutations of the IL2rg, RAG1 and RAG2 genes in in vitro developmental embryos by microinjected of Cas9 mRNA together with gRNAs for IL2rg, RAG1 and RAG2. (B) Sequenced mutations of the IL2rg, RAG1, RAG2, TIKI1 and ALB genes occurred in in vitro developmental embryos by microinjection of Cas9 mRNA together with gRNAs for IL2rg, RAG1, RAG2, TIKI1 and ALB. For each gene, the WT sequence is shown at the top with the target sites in underline. Deletions are indicated by dashes, insertions are indicated in blue and substitutions are indicated in pink, and the sizes of the deletions (-) or insertions (+) are shown on the right column. (PPTX 85 KB) [file 13619_2014_27_MOESM1_ESM.pptx]

## Slide 1
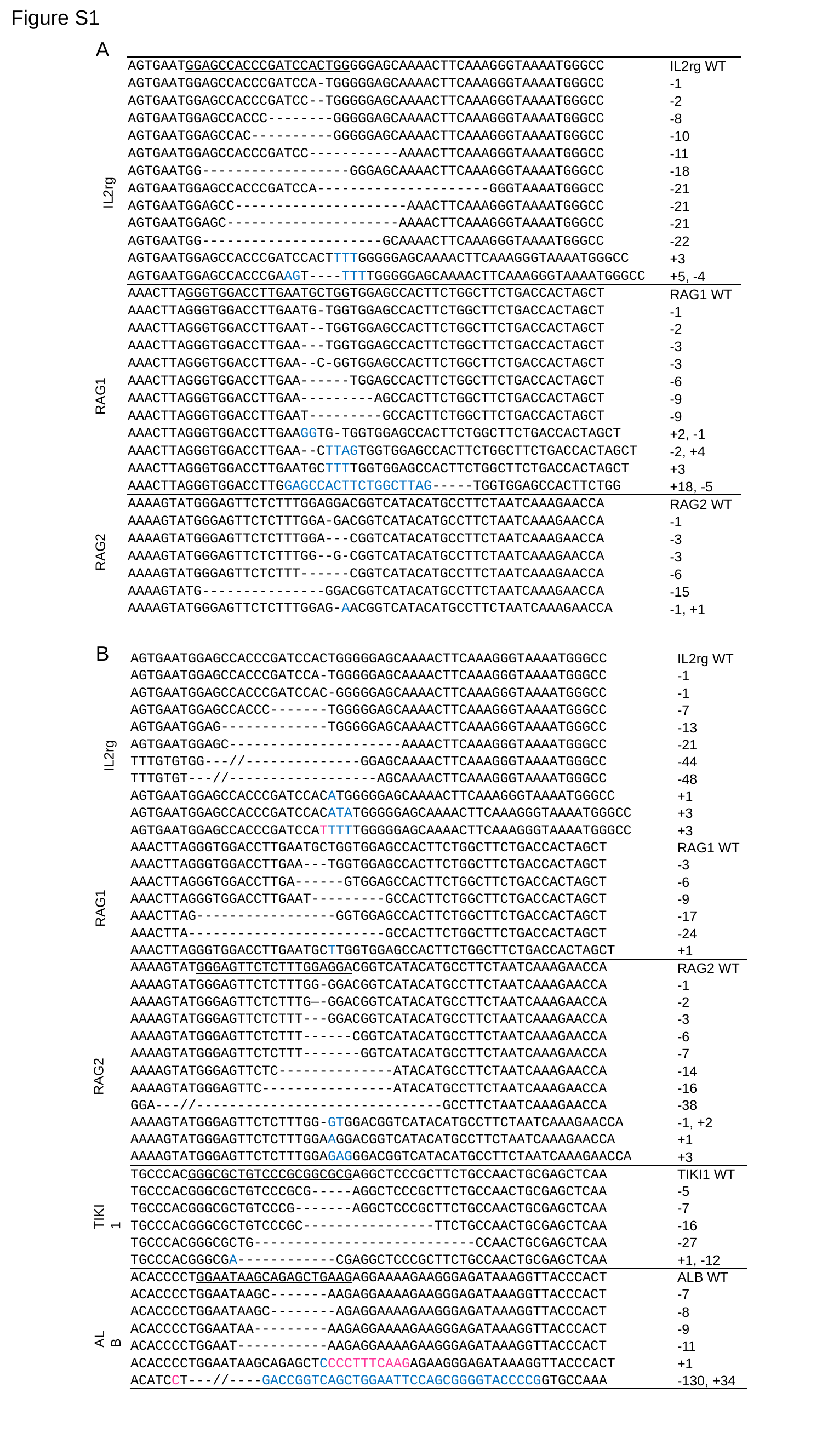

Figure S1
A
| AGTGAATGGAGCCACCCGATCCACTGGGGGAGCAAAACTTCAAAGGGTAAAATGGGCC | IL2rg WT |
| --- | --- |
| AGTGAATGGAGCCACCCGATCCA-TGGGGGAGCAAAACTTCAAAGGGTAAAATGGGCC | -1 |
| AGTGAATGGAGCCACCCGATCC--TGGGGGAGCAAAACTTCAAAGGGTAAAATGGGCC | -2 |
| AGTGAATGGAGCCACCC--------GGGGGAGCAAAACTTCAAAGGGTAAAATGGGCC | -8 |
| AGTGAATGGAGCCAC----------GGGGGAGCAAAACTTCAAAGGGTAAAATGGGCC | -10 |
| AGTGAATGGAGCCACCCGATCC-----------AAAACTTCAAAGGGTAAAATGGGCC | -11 |
| AGTGAATGG------------------GGGAGCAAAACTTCAAAGGGTAAAATGGGCC | -18 |
| AGTGAATGGAGCCACCCGATCCA---------------------GGGTAAAATGGGCC | -21 |
| AGTGAATGGAGCC---------------------AAACTTCAAAGGGTAAAATGGGCC | -21 |
| AGTGAATGGAGC---------------------AAAACTTCAAAGGGTAAAATGGGCC | -21 |
| AGTGAATGG----------------------GCAAAACTTCAAAGGGTAAAATGGGCC | -22 |
| AGTGAATGGAGCCACCCGATCCACTTTTGGGGGAGCAAAACTTCAAAGGGTAAAATGGGCC | +3 |
| AGTGAATGGAGCCACCCGAAGT----TTTTGGGGGAGCAAAACTTCAAAGGGTAAAATGGGCC | +5, -4 |
| AAACTTAGGGTGGACCTTGAATGCTGGTGGAGCCACTTCTGGCTTCTGACCACTAGCT | RAG1 WT |
| AAACTTAGGGTGGACCTTGAATG-TGGTGGAGCCACTTCTGGCTTCTGACCACTAGCT | -1 |
| AAACTTAGGGTGGACCTTGAAT--TGGTGGAGCCACTTCTGGCTTCTGACCACTAGCT | -2 |
| AAACTTAGGGTGGACCTTGAA---TGGTGGAGCCACTTCTGGCTTCTGACCACTAGCT | -3 |
| AAACTTAGGGTGGACCTTGAA--C-GGTGGAGCCACTTCTGGCTTCTGACCACTAGCT | -3 |
| AAACTTAGGGTGGACCTTGAA------TGGAGCCACTTCTGGCTTCTGACCACTAGCT | -6 |
| AAACTTAGGGTGGACCTTGAA---------AGCCACTTCTGGCTTCTGACCACTAGCT | -9 |
| AAACTTAGGGTGGACCTTGAAT---------GCCACTTCTGGCTTCTGACCACTAGCT | -9 |
| AAACTTAGGGTGGACCTTGAAGGTG-TGGTGGAGCCACTTCTGGCTTCTGACCACTAGCT | +2, -1 |
| AAACTTAGGGTGGACCTTGAA--CTTAGTGGTGGAGCCACTTCTGGCTTCTGACCACTAGCT | -2, +4 |
| AAACTTAGGGTGGACCTTGAATGCTTTTGGTGGAGCCACTTCTGGCTTCTGACCACTAGCT | +3 |
| AAACTTAGGGTGGACCTTGGAGCCACTTCTGGCTTAG-----TGGTGGAGCCACTTCTGG | +18, -5 |
| AAAAGTATGGGAGTTCTCTTTGGAGGACGGTCATACATGCCTTCTAATCAAAGAACCA | RAG2 WT |
| AAAAGTATGGGAGTTCTCTTTGGA-GACGGTCATACATGCCTTCTAATCAAAGAACCA | -1 |
| AAAAGTATGGGAGTTCTCTTTGGA---CGGTCATACATGCCTTCTAATCAAAGAACCA | -3 |
| AAAAGTATGGGAGTTCTCTTTGG--G-CGGTCATACATGCCTTCTAATCAAAGAACCA | -3 |
| AAAAGTATGGGAGTTCTCTTT------CGGTCATACATGCCTTCTAATCAAAGAACCA | -6 |
| AAAAGTATG---------------GGACGGTCATACATGCCTTCTAATCAAAGAACCA | -15 |
| AAAAGTATGGGAGTTCTCTTTGGAG-AACGGTCATACATGCCTTCTAATCAAAGAACCA | -1, +1 |
IL2rg
RAG1
RAG2
B
| AGTGAATGGAGCCACCCGATCCACTGGGGGAGCAAAACTTCAAAGGGTAAAATGGGCC | IL2rg WT |
| --- | --- |
| AGTGAATGGAGCCACCCGATCCA-TGGGGGAGCAAAACTTCAAAGGGTAAAATGGGCC | -1 |
| AGTGAATGGAGCCACCCGATCCAC-GGGGGAGCAAAACTTCAAAGGGTAAAATGGGCC | -1 |
| AGTGAATGGAGCCACCC-------TGGGGGAGCAAAACTTCAAAGGGTAAAATGGGCC | -7 |
| AGTGAATGGAG-------------TGGGGGAGCAAAACTTCAAAGGGTAAAATGGGCC | -13 |
| AGTGAATGGAGC---------------------AAAACTTCAAAGGGTAAAATGGGCC | -21 |
| TTTGTGTGG---//--------------GGAGCAAAACTTCAAAGGGTAAAATGGGCC | -44 |
| TTTGTGT---//------------------AGCAAAACTTCAAAGGGTAAAATGGGCC | -48 |
| AGTGAATGGAGCCACCCGATCCACATGGGGGAGCAAAACTTCAAAGGGTAAAATGGGCC | +1 |
| AGTGAATGGAGCCACCCGATCCACATATGGGGGAGCAAAACTTCAAAGGGTAAAATGGGCC | +3 |
| AGTGAATGGAGCCACCCGATCCATTTTTGGGGGAGCAAAACTTCAAAGGGTAAAATGGGCC | +3 |
| AAACTTAGGGTGGACCTTGAATGCTGGTGGAGCCACTTCTGGCTTCTGACCACTAGCT | RAG1 WT |
| AAACTTAGGGTGGACCTTGAA---TGGTGGAGCCACTTCTGGCTTCTGACCACTAGCT | -3 |
| AAACTTAGGGTGGACCTTGA------GTGGAGCCACTTCTGGCTTCTGACCACTAGCT | -6 |
| AAACTTAGGGTGGACCTTGAAT---------GCCACTTCTGGCTTCTGACCACTAGCT | -9 |
| AAACTTAG-----------------GGTGGAGCCACTTCTGGCTTCTGACCACTAGCT | -17 |
| AAACTTA------------------------GCCACTTCTGGCTTCTGACCACTAGCT | -24 |
| AAACTTAGGGTGGACCTTGAATGCTTGGTGGAGCCACTTCTGGCTTCTGACCACTAGCT | +1 |
| AAAAGTATGGGAGTTCTCTTTGGAGGACGGTCATACATGCCTTCTAATCAAAGAACCA | RAG2 WT |
| AAAAGTATGGGAGTTCTCTTTGG-GGACGGTCATACATGCCTTCTAATCAAAGAACCA | -1 |
| AAAAGTATGGGAGTTCTCTTTG—-GGACGGTCATACATGCCTTCTAATCAAAGAACCA | -2 |
| AAAAGTATGGGAGTTCTCTTT---GGACGGTCATACATGCCTTCTAATCAAAGAACCA | -3 |
| AAAAGTATGGGAGTTCTCTTT------CGGTCATACATGCCTTCTAATCAAAGAACCA | -6 |
| AAAAGTATGGGAGTTCTCTTT-------GGTCATACATGCCTTCTAATCAAAGAACCA | -7 |
| AAAAGTATGGGAGTTCTC--------------ATACATGCCTTCTAATCAAAGAACCA | -14 |
| AAAAGTATGGGAGTTC----------------ATACATGCCTTCTAATCAAAGAACCA | -16 |
| GGA---//------------------------------GCCTTCTAATCAAAGAACCA | -38 |
| AAAAGTATGGGAGTTCTCTTTGG-GTGGACGGTCATACATGCCTTCTAATCAAAGAACCA | -1, +2 |
| AAAAGTATGGGAGTTCTCTTTGGAAGGACGGTCATACATGCCTTCTAATCAAAGAACCA | +1 |
| AAAAGTATGGGAGTTCTCTTTGGAGAGGGACGGTCATACATGCCTTCTAATCAAAGAACCA | +3 |
| TGCCCACGGGCGCTGTCCCGCGGCGCGAGGCTCCCGCTTCTGCCAACTGCGAGCTCAA | TIKI1 WT |
| TGCCCACGGGCGCTGTCCCGCG-----AGGCTCCCGCTTCTGCCAACTGCGAGCTCAA | -5 |
| TGCCCACGGGCGCTGTCCCG-------AGGCTCCCGCTTCTGCCAACTGCGAGCTCAA | -7 |
| TGCCCACGGGCGCTGTCCCGC----------------TTCTGCCAACTGCGAGCTCAA | -16 |
| TGCCCACGGGCGCTG---------------------------CCAACTGCGAGCTCAA | -27 |
| TGCCCACGGGCGA------------CGAGGCTCCCGCTTCTGCCAACTGCGAGCTCAA | +1, -12 |
| ACACCCCTGGAATAAGCAGAGCTGAAGAGGAAAAGAAGGGAGATAAAGGTTACCCACT | ALB WT |
| ACACCCCTGGAATAAGC-------AAGAGGAAAAGAAGGGAGATAAAGGTTACCCACT | -7 |
| ACACCCCTGGAATAAGC--------AGAGGAAAAGAAGGGAGATAAAGGTTACCCACT | -8 |
| ACACCCCTGGAATAA---------AAGAGGAAAAGAAGGGAGATAAAGGTTACCCACT | -9 |
| ACACCCCTGGAAT-----------AAGAGGAAAAGAAGGGAGATAAAGGTTACCCACT | -11 |
| ACACCCCTGGAATAAGCAGAGCTCCCCTTTCAAGAGAAGGGAGATAAAGGTTACCCACT | +1 |
| ACATCCT---//----GACCGGTCAGCTGGAATTCCAGCGGGGTACCCCGGTGCCAAA | -130, +34 |
IL2rg
RAG1
RAG2
TIKI1
ALB
