# Supplementary material for: Generation of multi-gene knockout rabbits using the Cas9/gRNA system
Source: Cell Regen. 2014 Sep 27;3:12. doi: 10.1186/2045-9769-3-12 (PMC4230364; doi:10.1186/2045-9769-3-12)
Supplement: Supplementary file 2 — Additional file 2: Figure S2: Candidate off-target sites are presented in terms of chromosome location, sequence, overall percent match with the targets (mismatches are indicated in red) and expected amplicon and T7EI fragment sizes. (PPTX 113 KB) [file 13619_2014_27_MOESM2_ESM.pptx]

## Slide 1
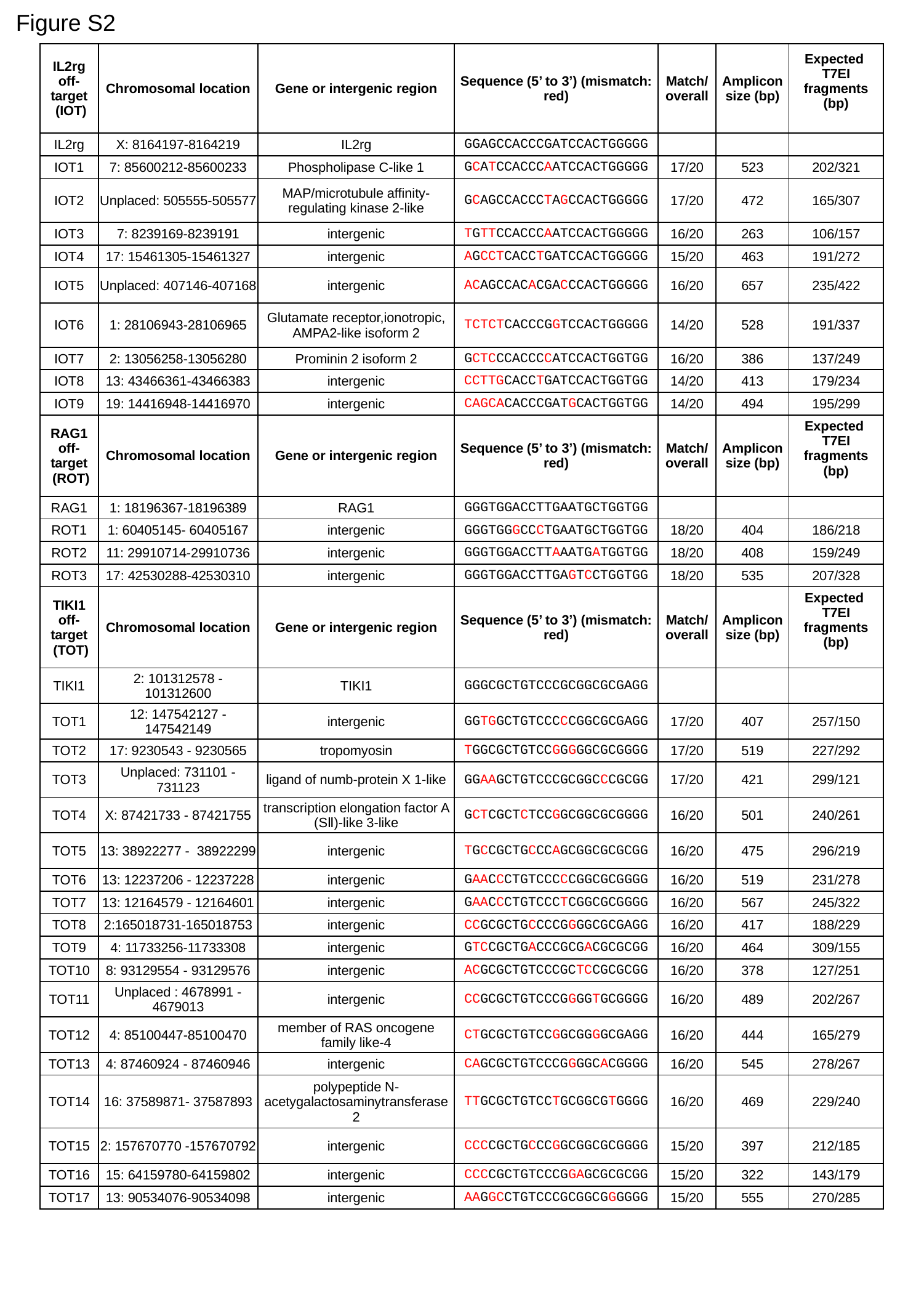

Figure S2
| IL2rg off-target (IOT) | Chromosomal location | Gene or intergenic region | Sequence (5’ to 3’) (mismatch: red) | Match/ overall | Amplicon size (bp) | Expected T7EI fragments (bp) |
| --- | --- | --- | --- | --- | --- | --- |
| IL2rg | X: 8164197-8164219 | IL2rg | GGAGCCACCCGATCCACTGGGGG | | | |
| IOT1 | 7: 85600212-85600233 | Phospholipase C-like 1 | GCATCCACCCAATCCACTGGGGG | 17/20 | 523 | 202/321 |
| IOT2 | Unplaced: 505555-505577 | MAP/microtubule affinity-regulating kinase 2-like | GCAGCCACCCTAGCCACTGGGGG | 17/20 | 472 | 165/307 |
| IOT3 | 7: 8239169-8239191 | intergenic | TGTTCCACCCAATCCACTGGGGG | 16/20 | 263 | 106/157 |
| IOT4 | 17: 15461305-15461327 | intergenic | AGCCTCACCTGATCCACTGGGGG | 15/20 | 463 | 191/272 |
| IOT5 | Unplaced: 407146-407168 | intergenic | ACAGCCACACGACCCACTGGGGG | 16/20 | 657 | 235/422 |
| IOT6 | 1: 28106943-28106965 | Glutamate receptor,ionotropic, AMPA2-like isoform 2 | TCTCTCACCCGGTCCACTGGGGG | 14/20 | 528 | 191/337 |
| IOT7 | 2: 13056258-13056280 | Prominin 2 isoform 2 | GCTCCCACCCCATCCACTGGTGG | 16/20 | 386 | 137/249 |
| IOT8 | 13: 43466361-43466383 | intergenic | CCTTGCACCTGATCCACTGGTGG | 14/20 | 413 | 179/234 |
| IOT9 | 19: 14416948-14416970 | intergenic | CAGCACACCCGATGCACTGGTGG | 14/20 | 494 | 195/299 |
| RAG1 off-target (ROT) | Chromosomal location | Gene or intergenic region | Sequence (5’ to 3’) (mismatch: red) | Match/ overall | Amplicon size (bp) | Expected T7EI fragments (bp) |
| RAG1 | 1: 18196367-18196389 | RAG1 | GGGTGGACCTTGAATGCTGGTGG | | | |
| ROT1 | 1: 60405145- 60405167 | intergenic | GGGTGGGCCCTGAATGCTGGTGG | 18/20 | 404 | 186/218 |
| ROT2 | 11: 29910714-29910736 | intergenic | GGGTGGACCTTAAATGATGGTGG | 18/20 | 408 | 159/249 |
| ROT3 | 17: 42530288-42530310 | intergenic | GGGTGGACCTTGAGTCCTGGTGG | 18/20 | 535 | 207/328 |
| TIKI1 off-target (TOT) | Chromosomal location | Gene or intergenic region | Sequence (5’ to 3’) (mismatch: red) | Match/ overall | Amplicon size (bp) | Expected T7EI fragments (bp) |
| TIKI1 | 2: 101312578 - 101312600 | TIKI1 | GGGCGCTGTCCCGCGGCGCGAGG | | | |
| TOT1 | 12: 147542127 - 147542149 | intergenic | GGTGGCTGTCCCCCGGCGCGAGG | 17/20 | 407 | 257/150 |
| TOT2 | 17: 9230543 - 9230565 | tropomyosin | TGGCGCTGTCCGGGGGCGCGGGG | 17/20 | 519 | 227/292 |
| TOT3 | Unplaced: 731101 - 731123 | ligand of numb-protein X 1-like | GGAAGCTGTCCCGCGGCCCGCGG | 17/20 | 421 | 299/121 |
| TOT4 | X: 87421733 - 87421755 | transcription elongation factor A (SⅡ)-like 3-like | GCTCGCTCTCCGGCGGCGCGGGG | 16/20 | 501 | 240/261 |
| TOT5 | 13: 38922277 - 38922299 | intergenic | TGCCGCTGCCCAGCGGCGCGCGG | 16/20 | 475 | 296/219 |
| TOT6 | 13: 12237206 - 12237228 | intergenic | GAACCCTGTCCCCCGGCGCGGGG | 16/20 | 519 | 231/278 |
| TOT7 | 13: 12164579 - 12164601 | intergenic | GAACCCTGTCCCTCGGCGCGGGG | 16/20 | 567 | 245/322 |
| TOT8 | 2:165018731-165018753 | intergenic | CCGCGCTGCCCCGGGGCGCGAGG | 16/20 | 417 | 188/229 |
| TOT9 | 4: 11733256-11733308 | intergenic | GTCCGCTGACCCGCGACGCGCGG | 16/20 | 464 | 309/155 |
| TOT10 | 8: 93129554 - 93129576 | intergenic | ACGCGCTGTCCCGCTCCGCGCGG | 16/20 | 378 | 127/251 |
| TOT11 | Unplaced : 4678991 - 4679013 | intergenic | CCGCGCTGTCCCGGGGTGCGGGG | 16/20 | 489 | 202/267 |
| TOT12 | 4: 85100447-85100470 | member of RAS oncogene family like-4 | CTGCGCTGTCCGGCGGGGCGAGG | 16/20 | 444 | 165/279 |
| TOT13 | 4: 87460924 - 87460946 | intergenic | CAGCGCTGTCCCGGGGCACGGGG | 16/20 | 545 | 278/267 |
| TOT14 | 16: 37589871- 37587893 | polypeptide N-acetygalactosaminytransferase 2 | TTGCGCTGTCCTGCGGCGTGGGG | 16/20 | 469 | 229/240 |
| TOT15 | 2: 157670770 -157670792 | intergenic | CCCCGCTGCCCGGCGGCGCGGGG | 15/20 | 397 | 212/185 |
| TOT16 | 15: 64159780-64159802 | intergenic | CCCCGCTGTCCCGGAGCGCGCGG | 15/20 | 322 | 143/179 |
| TOT17 | 13: 90534076-90534098 | intergenic | AAGGCCTGTCCCGCGGCGGGGGG | 15/20 | 555 | 270/285 |
