# Supplementary material for: Generation of multi-gene knockout rabbits using the Cas9/gRNA system
Source: Cell Regen. 2014 Sep 27;3:12. doi: 10.1186/2045-9769-3-12 (PMC4230364; doi:10.1186/2045-9769-3-12)
Supplement: Supplementary file 3 — Additional file 3: Table S1: Primer pairs used to amplify the fragments encompassing the targeted sites of IL2rg, RAG1, RAG2, TIKI1 and ALB. Table S2. Primer pairs used to amplify the fragments encompassing the candidate off-targeted sites of IL2rg, RAG1and TIKI1. (DOC 82 KB) [file 13619_2014_27_MOESM3_ESM.doc]

Table S1. Primer pairs used to amplify the fragments encompassing the targeted sites of IL2rg, RAG1, RAG2, TIKI1 and ALB.

| Primer | Sequence (5’-3’) |
| --- | --- |
| IL2rg-F1 | CCTGATGCCAGAGACACAAG |
| IL2rg-R1 | CCACTCCCCTACTCTGAAAATAC |
| IL2rg-F2 | GGAGGGAAGATCCAGAACTG |
| IL2rg-R2 | CAGAGCCGGAAGTGTGTTAC |
| IL2rg-R3 | CTTAACCAGTGGCACCGCAATA |
| RAG1-F1 | AATTTAAGCTATTCAGGGTGCG |
| RAG1-R1 | TTGGCTTGGTGGATTGCTTT |
| RAG1-F2 | CAAGGTTTTCCGGATTGATGT |
| RAG1-R2 | ATCCTTGCCTGAACCCTCCTC |
| RAG2-F1 | CCTTAATTCAACCAGGCTTCTCACT |
| RAG2-R1 | TTCTGGAACATCTCCTACCAAGTCT |
| RAG2-F2 | ATCCACGGAGGGAAAACACC |
| RAG2-R2 | TCTGGAAGAATGTAGGATGTGGC |
| TIKI1-F1 | CGCTACTTCGCCCATTCCC |
| TIKI1-R1 | GCAAAGTGCCCAAGGTCCAG |
| TIKI1-R2 | CGCTGCCGTGCGAGTGGAA |
| ALB-F1 | GAATTGGCAAGGTTTTGTACAGA |
| ALB-R1 | GATAATCTCATAAGGGCTCACAA |
| ALB-F2 | GCAATCAGTTGAGAGCCAATGA |

Table S2. Primer pairs used to amplify the fragments encompassing the candidate off-targeted sites of IL2rg, RAG1and TIKI1.

| Primer | Sequence (5’-3’) |
| --- | --- |
| IOT1-F | ACTGGGTGGAGGTACATGTCAAG |
| IOT1-R | ACTGGGTGGAGGTACATGTCAAG |
| IOT2-F | TTTCTTCAGGGAGATGTGAGGATT |
| IOT2-R | GTATGACTCTTATTTGCCAGTTATGC |
| IOT3-F | AGAGGGAATAGGAGGTGGGA |
| IOT3-R | CCTACCAGCCGGTGAGTCAT |
| IOT4-F | GGGGACCAGAACTTTGATGCT |
| IOT4-R | ACCCAACCAGAGGAACCACAC |
| IOT5-F | CTCACGTGTACCACCCTAAGCTC |
| IOT5-R | CCATCCTTGCCCTGTGATTTAT |
| IOT6-F | TCTCCCATTTGGCTACAGGTCC |
| IOT6-R | TCTGGAGAAAGAAAGCGGGTTG |
| IOT7-F | CACAGTGGACACGGGGCTAA |
| IOT7-R | CGTGGGCTTCAGCTTCCTCT |
| IOT8-F | TTTCCTATCTTGTTCAAAATAATCT |
| IOT8-R | TCTGAGGAAGCATCATCAAATAC |
| IOT9-F | TTTGTGCTGATCCTGTTCATTCCT |
| IOT9-R | GCCCAGACCTGACTGTTGTG |
| ROT1-F | TCCCCCTGCTGTGCCCTG |
| ROT1-R | GCTTCACTACCTTCTCCAGTAACCA |
| ROT2-F | GTTCTGCTATTTTCCAAGGTGCCGT |
| ROT2-R | TCTTCCTTATGGATCTAAAGCACTC |
| ROT3-F | CTCCTGGTTCAAAATGAGATGTG |
| ROT3-R | GTTAAATGTCTTGTTCTGCCTATGT |
| TOT1-F | ATTTGAGCCATCACTACTGCCTTC |
| TOT1-R | GGGACATCAGGTAGGAGGAGGT |
| TOT2-F | GCTGTGGCCAGGATTATAAGG |
| TOT2-R | CTTCTCTGGGCTTCAGCTATAAAAA |
| TOT3-F | GGCAGGCACCGTGTGTAGC |
| TOT3-R | AGCAGCGTGCCCCACAACTA |
| TOT4-F | CTCTGCATCCAGTGGAAACCG |
| TOT4-R | CTACCCCCAGCACAGGACAAG |
| TOT5-F | GGGGCTCCTTCTCTCCGACT |
| TOT5-R | TTTCCTCCCCCTGTTGGACTTG |
| TOT6-F | AAACACAACAGTAAGAAGGAAAGGC |
| TOT6-R | CGTTGCCCTCACCCGTTG |
| TOT7-F | CTCTCCAATGAATAAACACAACAGT |
| TOT7-R | ACGTGTGATCCCTGCACTTTC |
| TOT8-F | GCCCTGAAATCACCATTGAAGAC |
| TOT8-R | GAGAAGTCAGGAGGCTCTGTTGG |
| TOT9-F | GGCGTGAATCAACACACCTCT |
| TOT9-R | AGGTAGCGGCAGCGTTGTG |
| TOT10-F | GGAACATCCAGGTAGTAGGACAACA |
| TOT10-R | TTTTGCATCGTCCACAGATCCCAT |
| TOT11-F | GGGGTCTTCAAAAAGTCCACG |
| TOT11-R | CTGCTCCACACCGATACTGCC |
| TOT12-F | TCTTGTCCCTTCCCTTCCGC |
| TOT12-R | CCCCCCGACATCTAACGCT |
| TOT13-F | AGCAGGCACTCCCAGCAGA |
| TOT13-R | TTCAGAGACACCCTTTGGCGTAT |
| TOT14-F | TTGGTCTGTATGGAGCCTGGA |
| TOT14-R | TCATAGCATACTCGATTAGGGTTC |
| TOT15-F | CTGGAGTTTGTCTCTAACGACCGAT |
| TOT15-R | GGAAAATAATCACAGCCCTGAAGAC |
| TOT16-F | CGCCCGAAGCCTTATCCGA |
| TOT16-R | CCCTCCTTGTGTCTGGTTGCCT |
| TOT17-F | CCAATTTTCGGATCACAGTACG |
| TOT17-R | TTTTGACTCCTCCTTTTCTCCC |
